# Response Surface Methodology-Optimized Synthesis of ZIF-8 Nanoparticles and Its Application in the Extraction of Anthraquinones from Cassia Seed

**DOI:** 10.3390/mi17070774

**Published:** 2026-06-26

**Authors:** Chunhua Qu, Yafei Yang, Yan Liu, Guang Xu, Jing Zeng, Mengqin Li

**Affiliations:** College of Chinese Materia Medica, Chongqing University of Chinese Medicine, Chongqing 402760, China; yangyafei@cqctcm.edu.cn (Y.Y.); liuyantaney@126.com (Y.L.); xuguang@cqctcm.edu.cn (G.X.); zengxijing@163.com (J.Z.); zackink@163.com (M.L.)

**Keywords:** response surface methodology, solvothermal method, ZIF-8 nanoparticles, anthraquinones, cassia semen, UPLC

## Abstract

In this study, response surface methodology (RSM) was employed to evaluate and optimize the key parameters for the solvothermal synthesis of Zeolitic Imidazolate Framework-8 (ZIF-8) nanoparticles, including solvent type, reaction temperature, reaction time, and material ratio. A multivariate regression model identified the optimal preparation conditions as ethanol as the solvent, a reaction temperature of 120 °C, a reaction time of 4 h, and a 5:1 molar ratio of 2-methylimidazole to zinc acetate. The resulting ZIF-8 nanoparticles exhibited highly selective adsorption capacity toward anthraquinones and were successfully applied to the rapid extraction and detection of five anthraquinones from Cassiae semen. By investigating the adsorbent dosage, adsorption efficiency, elution solvent, and elution efficiency, we established the optimal experimental conditions. Briefly, 20 mg of ZIF-8 nanoparticles were added to 10 mL of Cassia semen extract, and the mixture was shaken for 10 min before centrifugation. The residual anthraquinones in the supernatant were quantified by Ultra Performance Liquid Chromatography (UPLC). The adsorption efficiencies of aloe-emodin, rhein, emodin, chrysophanol, and physcion were 80.2%, 93.8%, 100%, 100%, and 100%, respectively. When eluted with methanol/100 mM NaHCO_3_ solution (1:1, *v*/*v*), the corresponding elution efficiencies of these compounds were 82.8%, 97.8%, 85.1%, 93.2%, and 65.3%, respectively. The relative standard deviations (RSDs) for method precision, stability, and repeatability were all below 4.0%. The prepared ZIF-8 nanoparticles showed favorable adsorption performance toward the five anthraquinone components. The method is simple to operate, requires minimal sample and solvent consumption, and can be used for rapid extraction and detection of anthraquinones in traditional Chinese medicinal materials such as Cassiae semen. This work provides a scientific reference for the application of MOFs nanomaterials in food safety inspection and quality control of traditional Chinese medicinal materials.

## 1. Introduction

Anthraquinones, which exist in both conjugated and free aglycone forms, include aloe-emodin, rhein, emodin, chrysophanol and physcion. These compounds exhibit potent pharmacological activities, including anti-inflammatory and antibacterial effects [1,2,3], hypolipidemic and cholesterol-lowering Effects [4], antiplatelet aggregation, antioxidant abilities [5], antitumor efficacy [6,7], and anti-Hepatitis B virus activity [8]. Cassia semen, first documented in Shen Nong Ben Cao Jing, has a sweet, bitter, and salty taste and is mildly cold in nature. It functions to clear the liver, improve vision, and promote bowel movements. Anthraquinones are one of its primary bioactive constituents.

Various techniques have been developed for the extraction and detection of anthraquinones. For extraction, natural deep eutectic solvent extraction [9], matrix solid-phase dispersion [10], and microwave-assisted ionic liquid homogeneous liquid–liquid microextraction [11] have been commonly used. However, natural deep eutectic solvents often require prolonged extraction times and pose challenges in solvent recovery [12], matrix solid-phase dispersion is labor-intensive and may yield inconsistent recoveries depending on the sample matrix [13], while ionic liquid homogeneous liquid–liquid microextraction suffers from high viscosity, poor compatibility with HPLC, and significant sensitivity to ionic strength [14]. For detection, high performance capillary electrophoresis [15] and high-performance liquid chromatography (HPLC) [9,10,11] are the primary methods. Capillary electrophoresis offers high separation efficiency but suffers from limited sensitivity for trace analysis and susceptibility to matrix interference. Conventional HPLC, while reliable and widely used, typically involves long analysis times and high solvent consumption. To overcome the limitations of conventional extraction methods, we developed a solid-phase extraction method based on ZIF-8 nanoparticles for efficient sample pretreatment, coupled with UPLC as the detection technique, thereby improving detection sensitivity and reducing analysis time.

ZIF-8 is a zeolitic imidazolate framework, a subclass of metal-organic frameworks (MOFs), which are porous crystalline materials composed of metal ions or metal clusters connected to organic ligands through coordination bonds [16], characterized by high porosity, extensive surface area, tunable pore structures, abundant active sites, and favorable biocompatibility [17]. Based on the properties of active ingredients of traditional Chinese medicinal materials, MOFs were synthesized and modified to enable selective adsorption of specific drugs. This allows for the efficient and environmentally friendly extraction of active ingredients from Chinese medicinal materials. Researchers have progressively utilized MOFs for extracting target components from Chinese medicinal materials. For instance, Xiang and colleagues [18] synthesized a Cu(II)-based MOF effective for the extraction of quercetin (QT). Cui et al. [19] applied Zeolitic Imidazolate Framework-8 (ZIF-8) to isolate flavonoids from Caragana jubata. Zhang and coworkers [20] used MOF-assisted matrix solid-phase dispersion microextraction to separate five saponins from ginseng leaves. Chen et al. [21] developed novel covalent organic frameworks COF-based magnetic nanoparticles for the extraction of paclitaxel from rat plasma. Ma et al. [22] employed MOF-modified capillary columns to separate stereoisomers of pharmaceuticals, achieving successful isolation of ephedrine and pseudoephedrine. Zhou and colleagues [23] prepared magnetic metal-organic frameworks MIL-101(Fe) to extract three anthraquinones from rhubarb. These findings indicate that MOFs exhibit high adsorption capacity, highlighting their promise as effective materials for separation and purification applications.

Currently, the synthesis of MOFs remains technically demanding, typically requiring extended high-temperature calcination, which limits the rapid fabrication of materials with strong adsorption performance. Zeolitic Imidazolate Frameworks (ZIFs), a subclass of MOFs constructed with imidazolate linkers resembling zeolite structures, offer notable thermal and chemical robustness. Among them, ZIF-8 can be prepared quickly via a hydrothermal or solvothermal route that is both cost-effective and eco-friendly. ZIF-8 has been successfully applied to solid-phase extraction (SPE) and dispersive solid-phase extraction (d-SPE) for the determination of various analytes including pesticides [24], antibiotics [25], heavy metals [26], dyes [27], and hormones [28]. However, its application in the extraction of anthraquinones from traditional Chinese medicinal materials has not been reported so far.

In this work, a rapid solvothermal method was adopted to synthesize ZIF-8 for the selective extraction of five anthraquinones from Cassia semen. This represents the first application of ZIF-8 for the simultaneous extraction of five anthraquinones from this herbal matrix, significantly surpassing previous reports that focused on only one to three anthraquinones, primarily in biological fluids. Furthermore, unlike prior studies that often employed unoptimized or single-factor approaches, we systematically optimized the ZIF-8 synthesis using response surface methodology (RSM) tailored specifically for anthraquinone extraction, with a focus on traditional Chinese medicine quality control rather than pharmacokinetic applications. In fact, anthraquinones are common bioactive substances found not only in Cassia semen but also in other traditional Chinese herbs such as rhubarb, fleece-flower root, aloe, and senna leaf. Given their extensive distribution and significant pharmacological value, a universal and efficient pretreatment method for anthraquinones is urgently needed. Therefore, the proposed strategy is expected to improve the detection efficiency of bioactive constituents in Chinese medicinal materials, facilitate better quality control, and support further pharmacological investigations.

Conventional ZIF-8 synthesis methods, including room-temperature stirring [29], hydrothermal synthesis, and microwave-assisted synthesis have certain limitations. Among these, the room-temperature stirring method is simple to operate but often yields polydisperse particles with relatively low crystallinity. The hydrothermal method produces well-defined crystals but typically requires prolonged reaction times and high temperatures, resulting in high energy consumption [30]. Microwave-assisted synthesis is rapid but requires specialized equipment and suffers from poor scalability [31]. Early studies have mainly focused on optimizing individual parameters such as temperature, reaction time, and solvent ratio, while systematic investigations of multiple interacting variables remain limited. Therefore, a more comprehensive optimization strategy is urgently needed to simultaneously achieve desirable crystal morphology, high product yield, and excellent adsorption performance.

Response Surface Methodology (RSM) has been widely applied in the food industry to model and enhance processing operations by assessing how multiple variables and their interactions affect target responses [32,33,34,35,36]. In the present work, RSM was employed to examine the influence of several critical synthesis parameters on ZIF-8 preparation, including solvent selection, reaction temperature, reaction time, and material ratio. A Box–Behnken design was performed for optimization, leading to the successful fabrication of ZIF-8 nanoparticles with desirable morphology, high product yield, and strong adsorption capability. The morphology and adsorption properties of ZIF-8 were subsequently characterized by scanning electron microscopy (SEM), transmission electron microscopy (TEM), surface area analysis (BET), and ultra performance liquid chromatography (UPLC). This study not only provides a useful reference for the analysis of anthraquinones in natural products but also broadens the application of MOF-based solid-phase extraction (SPE) in the quality control of traditional Chinese medicinal materials and food safety inspection.

## 2. Materials and Methods

### 2.1. Chemicals and Materials

The reference compounds of aloe-emodin (Batch No. C15483497, 20 mg), rhein (Batch No. C15736624, 20 mg), emodin (Batch No. C12290988, 20 mg), chrysophanol (Batch No. C16987694, 20 mg) and physcion (Batch No. C17119771, 20 mg) were purchased from Shanghai Macklin Biochemical Technology Co., Ltd., Shanghai, China, all with purities exceeding 98%. 2-Methylimidazole and anhydrous zinc acetate were purchased from Shanghai Macklin Biochemical Technology Co., Ltd. HPLC-grade methanol was purchased from Honeywell Inc., Charlotte, NC, USA. Ultrapure water was prepared using an ultrapure water system from Sartorius Inc., Göttingen, Germany. Cassiae semen was purchased from a medicinal material wholesale market in Chongqing, China. All other reagents and chemicals used in this study were of analytical grade or higher.

### 2.2. Instrumentation

Reference compounds and samples were weighed using a Sartorius SQP analytical balance (0.01 mg readability, Sartorius, Germany) and an ME204E electronic analytical balance (Mettler-Toledo, Greifensee, Switzerland), respectively. Sample extraction and separation were performed using a CQ-400B-DST ultrasonic cleaner (Shanghai Yuejin Medical Instrument Co., Ltd., Shanghai, China), a Hallan M20 high-speed centrifuge (Hailang Yingke, Shanghai, China), and an Eppendorf 5425R refrigerated centrifuge (Eppendorf SE, Hamburg, Germany). Drying was carried out with an HZK-55 vacuum drying oven (Shanghai Yuejin Medical Instrument Co., Ltd., China). The SEM images of the prepared ZIF-8 were obtained using a ZEISS GeminiSEM 300 (Carl Zeiss, Jena, Germany). The TEM image was taken using a FEI Talos F200X G2 (Thermo Fisher Scientific, Waltham, MA, USA). Nitrogen sorption studies were carried out using a Micromeritics ASAP 2460 (Micromeritics, Norcross, GA, USA). The phase composition and framework formation of ZIF-8 were studied by a Rigaku SmartLab SE X-ray diffractometer (Rigaku Corporation, Tokyo, Japan) and a Thermo Scientific Nicolet iS20 Fourier transform infrared (FTIR) spectrometer (Thermo Fisher Scientific, USA). UPLC analysis was performed on a Thermo Scientific Vanquish Flex ultra-high-performance liquid chromatography system, equipped with a VF-D11 diode array detector, VF-P10 and VF-P20 binary/quaternary pumps, a VH-C10 column oven, a VF-A10 autosampler, and a Chromeleon 7.1 chromatography workstation (Thermo Fisher Scientific, USA).

### 2.3. Experimental Design Using Response Surface Methodology

Optimization of ZIF-8 synthesis was performed using a two-step strategy. First, solvent type was screened as a categorical variable using a one-factor-at-a-time approach, as it cannot be directly incorporated into the Box–Behnken design, which requires continuous numerical variables. After identifying the optimal solvent, the Box–Behnken design was applied to optimize the remaining continuous variables, including reaction temperature, material ratio, and reaction time. This two-step approach is efficient and widely adopted in MOF synthesis optimization.

Based on the single-factor optimization results, a Box–Behnken design (BBD) was employed to investigate the correlations between three independent variables and the adsorption efficiency of ZIF-8 toward five anthraquinones. Three key factors, including reaction temperature (A), material ratio (B), and reaction time (C) were selected as independent variables. A total of 17 experimental runs, including five replicates at the center point to evaluate the reproducibility of the extraction process. As shown in Table 1, three single factors that exhibited the greatest influence on the adsorption efficiency of ZIF-8 based on the results of single-factor experiments were selected along with three different levels (−1, 0, 1) for response surface design. The parameters of the model were estimated by the least square method based on 17 experimental runs of the Box–Behnken design, and the resulting model is presented in Table 2. The obtained test data were subjected to multiple regression fitting and process parameter optimization using Design-Expert 13.0 software. The optimal preparation conditions were subsequently validated and compared with the ideal values predicted by the software.

### 2.4. Preparation of ZIF-8 NPs

2-Methylimidazole (1.2316 g, 0.015 mol) was dissolved in 10 mL of ethanol to obtain a clear and transparent solution A. Anhydrous zinc acetate (0.5504 g, 0.003 mol) was dissolved in 20 mL of ethanol to obtain solution B. Then, solution B was slowly added to solution A under stirring to ensure uniform mixing. The resulting mixture was transferred into a 100 mL stainless steel autoclave lined with polytetrafluoroethylene (PTFE) and placed in a vacuum drying oven at 120 °C for 4 h. After the reaction was completed, the mixture was cooled to room temperature. The resulting white suspension was centrifuged at 9029× *g* for 15 min. After centrifugation, the supernatant was removed using a dropper pipette, and the precipitate was washed with 10 mL of ethanol 2–3 times. The washed precipitate was then vacuum-dried at 60 °C for 4 h to obtain ZIF-8 NPs. The as-prepared ZIF-8 nanoparticles were stored in a sealed desiccator at room temperature for subsequent extraction experiments.

### 2.5. Extraction and Detection of Five Anthraquinones

#### 2.5.1. Preparation of Standard Stock Solutions

Standard stock solutions of aloe-emodin, rhein, emodin, chrysophanol and physcion were prepared by accurately weighing 5.78, 4.83, 5.85, 5.37, and 4.51 mg of each reference standard, respectively. Each compound was dissolved in methanol in separate 50 mL beakers, quantitatively transferred to individual 50 mL volumetric flasks, and diluted to volume with methanol. The resulting stock solutions had concentrations of 115.6, 96.6, 117.0, 107.4, and 90.2 µg/mL, respectively. All stock solutions were stored at 4 °C.

#### 2.5.2. Preparation of Mixed Standard Solution

Accurately transfer 1.80 mL of each stock solution into a 10 mL volumetric flask, add methanol to volume and mix thoroughly to obtain a mixed standard solution containing the five anthraquinones.

#### 2.5.3. Extraction of Five Anthraquinones

Precisely transfer 1.00 mL of the mixed standard solution into a 10 mL volumetric flask, dilute to volume with 50% methanol, and mix to obtain the working solution. Accurately weigh 0.0100 g of the synthesized ZIF-8 NPs, add 1.00 mL of the working solution, shake for 10 min, and then centrifuge at 10,000× *g* for 10 min. The supernatant was collected, filtered through a 0.22 µm microporous membrane, and subjected to UPLC analysis. Subsequently, 1.00 mL of elution solvent (methanol/100 mM NaHCO_3_, 1:1, *v*/*v*) was added to the precipitate, followed by ultrasonication for 10 min. After centrifugation, the supernatant (eluent) was collected, filtered through a 0.22 µm microporous membrane, and analyzed by UPLC.

#### 2.5.4. Chromatographic Conditions

UPLC analysis was performed on a Thermal Scientific Vanquish Flex ultra-high-performance liquid chromatography system, using a Thermo Scientific Hypersil Gold Vanquish chromatographic column (150 mm × 2.1 mm, 1.9 µm) for separation. The mobile phase consisted of methanol (A) and 0.1% phosphoric acid aqueous solution (B), with the following gradient program: 0–3 min, solvent A increased from 80% to 85%; 3–5 min, maintained at 85% A; 5–6 min, A increased to 90%; 6–8 min, reverted to 80% A. The flow rate was set at 0.25 mL/min, injection volume was 1.0 µL and column temperature was maintained at 30 °C. The detection was performed using a diode array detector (DAD) at a wavelength of 254 nm.

## 3. Results and Discussion

### 3.1. Response Surface Analysis

The ultimate goal of this study was to obtain ZIF-8 nanoparticles with high adsorption efficiency toward anthraquinones for use in sample pretreatment. Therefore, the first step was to optimize the synthesis conditions of ZIF-8 in order to achieve high product yield, good crystallinity, and favorable surface area. In the optimization experiments, we employed a single-factor approach, investigating the effects of parameters such as solvent type, the molar ratio of reactants, reaction temperature, and reaction time. Based on this, the Box–Behnken response surface methodology was employed to focus on investigating the interactions between variables and to establish a quadratic regression model, ultimately predicting the global optimal synthesis conditions through the model. Subsequently, the ZIF-8 prepared under the optimized conditions was characterized, and its adsorption performance toward anthraquinones was evaluated.

**Figure 1:**
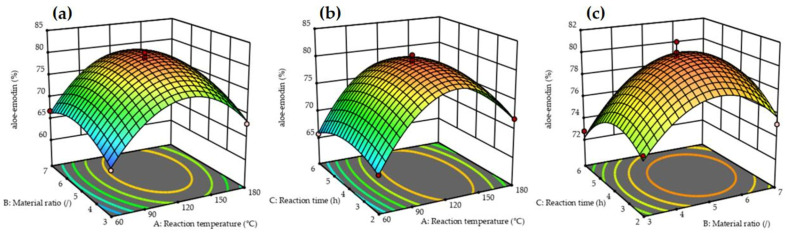
Multi-factor interaction 3D image of aloe-emodin. (**a**) The interaction of AB; (**b**) the interaction of AC; (**c**) the interaction of BC.

**Figure 2:**
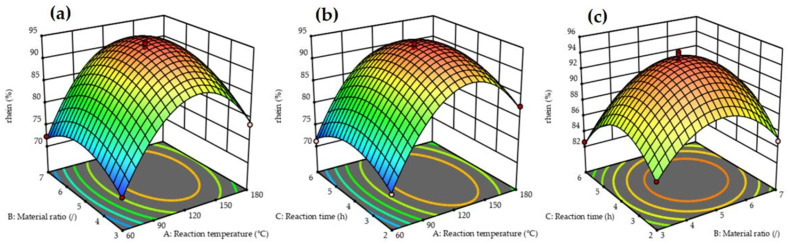
Multi-factor interaction 3D image of rhein. (**a**) The interaction of AB; (**b**) the interaction of AC; (**c**) the interaction of BC.

**Figure 3:**
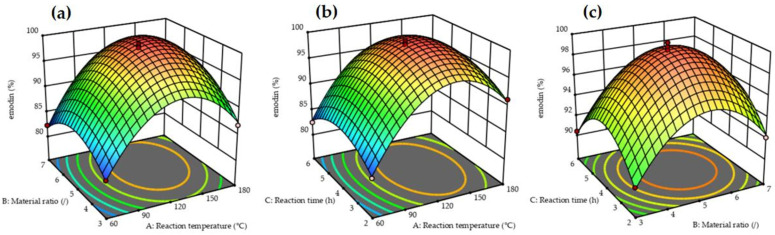
Multi-factor interaction 3D image of emodin. (**a**) The interaction of AB; (**b**) the interaction of AC; (**c**) the interaction of BC.

**Figure 4:**
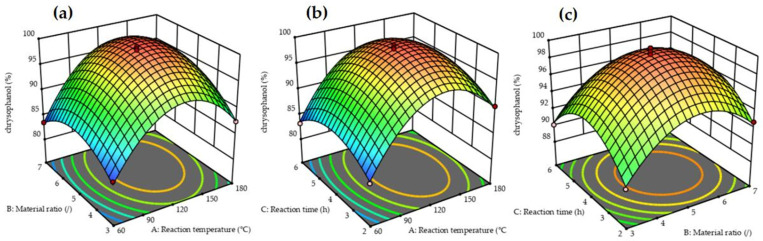
Multi-factor interaction 3D image of chrysophanol. (**a**) The interaction of AB; (**b**) the interaction of AC; (**c**) the interaction of BC.

**Figure 5:**
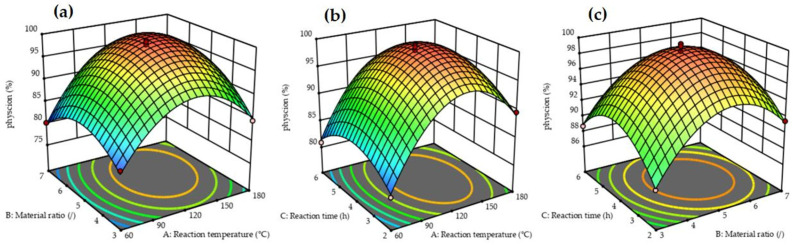
Multi-factor interaction 3D image of physcion. (**a**) The interaction of AB; (**b**) the interaction of AC; (**c**) the interaction of BC.

The 3D response surface and contour plots showing the effects of the interactions between various factors on the adsorption efficiencies of ZIF-8 toward five anthraquinones were generated using Design-Expert 13.0 software, as shown in Figure 1, Figure 2, Figure 3, Figure 4 and Figure 5. As can be seen from the response surface plot, with increasing reaction temperature, rising molar ratio of raw materials, and prolonged reaction time, the adsorption efficiencies of the five anthraquinones using the synthesized ZIF-8 were significantly enhanced, which is consistent with expectations. Therefore, based on the RSM analysis results, the optimal conditions for the synthesis of ZIF-8 were determined as follows: reaction temperature of 120 °C; reaction time of 4 h; and molar ratio of 2-methylimidazole to zinc acetate of 5:1.

The results of the analysis of variance (ANOVA) used to evaluate the model applicability among different factors and the suitability of the selected quadratic model are summarized in Table 3. The statistical significance of each polynomial equation was verified by means of *F*-tests and *p*-values. As shown in Table 3, the ANOVA for the second order response surface model of adsorption efficiencies of five anthraquinones demonstrated that the experimental data were highly significant, with all *p*-values being lower than 0.0001. Moreover, the *p*-values for the lack of fit were insignificant (0.3375–1.20) that confirming the assumption of the constant variance and validity of the applied models. The value of correlation coefficient R^2^ was 0.9878, 0.9952, 0.9961, 0.9940, and 0.9924 for the adsorption efficiencies of aloe-emodin, rhein, emodin, chrysophanol, and physcion, respectively, which suggested that the experimental data were fitted well with the models. Additionally, the linear terms (A, B, and C) and the quadratic terms (A^2^, B^2^, and C^2^) on adsorption efficiencies of five anthraquinones were significant (*p* < 0.05). In addition to this, t the interaction terms AB were found to be significant (*p* < 0.05) for the adsorption efficiencies of emodin, chrysophanol, and physcion, indicating that the interaction between reaction temperature and reaction time had the most significant effect. Consequently, this model can reliably reflect the relationship between various factors and the response value during the ZIF-8 preparation process, and can be used to predict the optimal process conditions.

The significant effects of reaction temperature, molar ratio of 2-methylimidazole to zinc acetate, and reaction time on extraction performance can be rationalized by the established crystallization pathway of ZIF-8. According to Venna et al. [37], ZIF-8 formation proceeds through nucleation, growth, and stationary phases, governed by solution- and solid-mediated transformation mechanisms with Avrami-type kinetics. Within this framework, elevated temperature accelerates coordination kinetics and deprotonation, promoting framework crystallinity; an appropriate excess of the organic linker ensures complete Zn–N coordination and stabilizes the sodalite-type structure; and prolonged reaction time allows for the consumption of metastable amorphous precursors and enables Ostwald ripening, yielding fully crystalline ZIF-8 with maximized surface area and accessible adsorption sites. Thus, the RSM-optimized conditions (120 °C, 4 h, and a 5:1 molar ratio of 2-methylimidazole to anhydrous zinc acetate) collectively ensure the completion of crystallization, consistent with both the statistical model and the mechanistic understanding of ZIF-8 formation.

To validate these optimized conditions, Design-Expert 13.0 was used to predict the ideal synthesis parameters based on the experimental data. The predicted adsorption efficiencies for aloe-emodin, rhein, emodin, chrysophanol, and physcion were 80.2%, 93.8%, 100%, 100%, and 100%, respectively. Subsequent experimental verification under the same conditions produced adsorption efficiencies that were in good agreement with the model predictions, confirming that the RSM model is reliable and suitable for optimizing the preparation process of ZIF-8.

### 3.2. Characterization of ZIF-8 NPs

**Figure 6:**
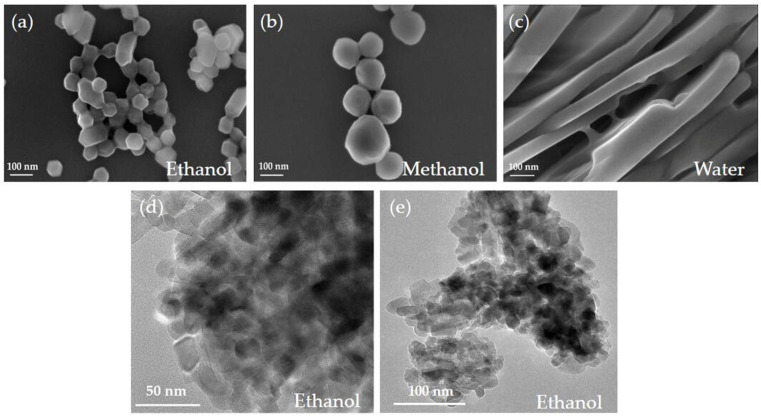
Characterizations of ZIF-8. (**a**) SEM image of ZIF-8 prepared in ethanol; (**b**) SEM image of ZIF-8 prepared in methanol; (**c**) SEM image of ZIF-8 prepared in water; (**d**,**e**) TEM image of ZIF-8 prepared in ethanol.

The morphology and structural features of the synthesized ZIF-8 nanoparticles were characterized using SEM and TEM, and the obtained images are shown in Figure 6. As can be seen from Figure 6a,b, the samples prepared in ethanol and methanol both exhibited a homogeneous particle morphology, with grain sizes falling in the range of approximately 50 to 150 nm. In contrast, the sample synthesized in water displayed a tendency to stack and form agglomerates (Figure 6c). During the co-crystallization process between 2-methylimidazole and anhydrous zinc acetate, Zn(II) ions coordinate with 2-methylimidazole molecules, triggering spontaneous self-assembly and the generation of ZIF-8 crystals with regular geometric shapes. The rates of crystal nucleation and growth are mainly controlled by the choice of solvent, the concentration of soluble species, and the reaction temperature. The solvothermal synthesis carried out in ethanol resulted in superior structural characteristics and a smaller particle size.

Figure 6d,e present higher-resolution TEM images, revealing that the synthesized ZIF-8 material exhibits a rhombic dodecahedral morphology, which is a typical characteristic feature of ZIF-8 crystals. The particles show smooth surfaces and sharply defined edges, indicating good crystallinity. This observed morphological characteristic is in good agreement with the results reported in reference [38], further confirming that ZIF-8 crystals have been successfully synthesized.

**Figure 7:**
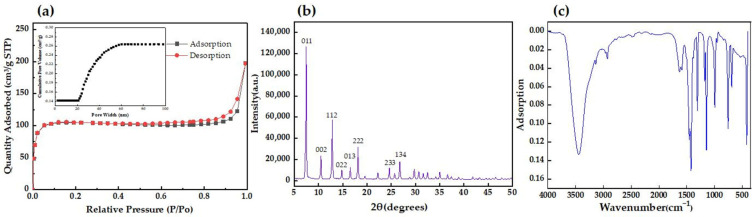
Characterizations of ZIF-8. (**a**) N_2_ adsorption–desorption isotherms and pore size distribution of ZIF-8; (**b**) X-ray powder diffraction pattern; (**c**) FTIR spectra.

To examine the structural properties of the ZIF-8 NPs, N_2_ adsorption–desorption measurements were performed at 77 K. As illustrated in Figure 7a, the samples exhibit a typical type I isotherm accompanied by a H1 hysteresis loop, revealing the coexistence of both microporous and mesoporous structures. Based on the single-point BET method, the total pore volume for pores with diameters smaller than 262.9 nm was calculated to be 0.30533 cm^3^/g at a relative pressure (P/P_0_) of 0.9927. The BET surface area of ZIF-8 was determined to be 375.89 m^2^/g, and the average pore diameter (derived from 4V/A by BET) was 3.25 nm. The high specific surface area and well-developed porous architecture of this material are expected to enhance its adsorption performance, thereby promoting the uptake and extraction of small-molecule compounds.

The measured surface area of our ZIF-8 is lower than the theoretical value (~1300 m^2^/g), as reported in some literature [39]. This discrepancy can be reasonably attributed to a combination of factors related to our specific synthesis conditions and material characteristics, including incomplete activation (e.g., residual solvent or unreacted precursors trapped within the pores), partial framework defects, or nanoparticle aggregation. As evidenced by the supplementary FTIR spectra presented in Figure 7c, the broad strong absorption band centered at approximately 3500 cm^−1^ is typical of O–H stretching vibrations, suggesting incomplete drying of the sample and the presence of considerable adsorbed water on the surface or within the pore channels. Nevertheless, as illustrated in Figure 7a, the samples exhibit a typical type I isotherm accompanied by an H1 hysteresis loop, revealing the coexistence of both microporous and mesoporous structures. This hierarchical porosity is expected to facilitate mass transfer and enhance the accessibility of target molecules, thereby promoting the uptake and extraction of small-molecule anthraquinones despite the moderate BET surface area.

The crystal structure and phase purity of the synthesized ZIF-8 were confirmed by powder X-ray diffraction (XRD). As shown in Figure 7b, the XRD peaks agree with those of typical ZIF-8 crystals. The peak position and characteristic peaks for ZIF-8 were observed to 7.3° (011), 10.3° (002), 12.7° (112), 14.7° (022), 16.4° (013), 18.0° (222), 24.6° (233) and 26.7° (134), which match well with the simulated pattern of ZIF-8 reported in the literature [37].

The FTIR spectrum shown in Figure 7c enabled the identification of the organic ligands in ZIF-8 and further confirmed the formation of the ZIF-8 framework. Characteristic absorption bands were observed at 1630 cm^−1^ (C=N stretching vibration of the imidazole ring), 1423 cm^−1^ (C–N stretching vibration of the imidazole ring), 1307 cm^−1^ (in-plane C–H bending vibration of the imidazole ring), 1144 cm^−1^ (out-of-plane C–H bending vibration of the imidazole ring), and 420 cm^−1^ (Zn–N stretching vibration).

### 3.3. Adsorption and Elution

In this study, the optimization of adsorption and desorption efficiency of the synthesized ZIF-8 was conducted using a one-factor-at-a-time approach. This decision was based on the following considerations. First, the extraction of anthraquinones from the solid matrix (Cassia semen) is a mass transfer process in which the effects of individual parameters (e.g., extraction time, solvent volume, pH) are largely independent, exhibiting relatively weak interactions compared to the nucleation and crystallization processes in MOF synthesis. Second, the one-factor-at-a-time method is straightforward and intuitive, allowing clear visualization of the trend for each variable, and is therefore widely used for solid–liquid extraction optimization of natural products. Third, preliminary experiments indicated that temperature and sonication regimes had only minor effects on recovery under our conditions. Consequently, we focused only on key parameters including adsorbent dosage, extraction time, and elution solvent.

#### 3.3.1. Selection of Adsorbent Dosage

**Figure 8:**
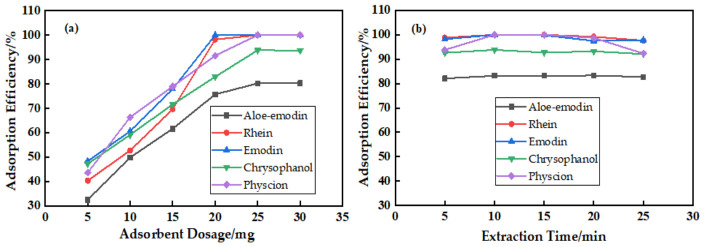
Influence of adsorbent dosage and extraction time on the adsorption efficiency of anthraquinone compounds: (**a**) Adsorbent dosage, (**b**) extraction time.

The influence of adsorbent dosage on adsorption efficiency was investigated. As the amount of adsorbent increased from 5.0 mg to 20.0 mg, the adsorption efficiency of anthraquinones showed a progressive rise. However, further increasing the dosage to 30.0 mg resulted in no significant improvement. With an adsorbent dosage of 20.0 mg, the adsorption efficiencies for aloe-emodin, rhein, emodin, chrysophanol, and physcion reached 80.2%, 93.8%, 100%, 100%, and 100%, respectively, all exceeding 80%. As illustrated in Figure 8a, a ZIF-8 dosage of 20.0 mg was identified as the optimal level for anthraquinones adsorption.

#### 3.3.2. Examination of Extraction Time

This study examined the impact of extraction time on the adsorption efficiency in the process of ZIF-8 NPs adsorbing five types of anthraquinones. Following the addition of ZIF-8 NPs to a mixed standard solution, the mixture was shaken for varying durations of 5, 10, 15, 20, and 25 min. The resulting data are presented in Figure 8b. The extraction time was observed to have only a minor effect on the adsorption efficiency of the anthraquinone compounds, with the highest efficiency achieved after 10 min. This indicates that the adsorption rate is very fast.

#### 3.3.3. The Selection of Elution Solvent

**Figure 9:**
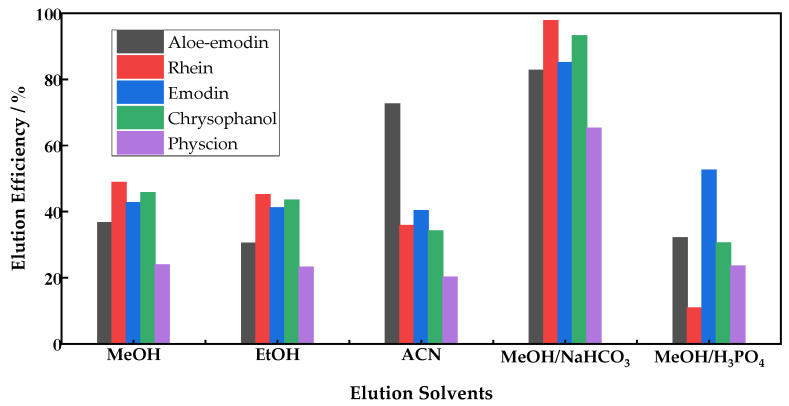
The influence of different solvents on elution efficiency.

The elution efficiencies of five solvents were evaluated, including methanol (MeOH), ethanol (EtOH), acetonitrile (ACN), methanol/100 mM NaHCO_3_ solution (1/1, MeOH/NaHCO_3_), and methanol/0.1% H_3_PO_4_ solution (1/1, MeOH/H_3_PO_4_). As shown in Figure 9, methanol and ethanol exhibited superior performance to acetonitrile in eluting rhein, chrysophanol, and physcion. In contrast, acetonitrile was more effective for the elution of aloe-emodin. Additionally, the 100 mM NaHCO_3_ solution demonstrated higher elution efficiency than the 0.1% H_3_PO_4_ solution. Based on the overall performance of these five solvents, the methanol/100 mM NaHCO_3_ solution (1/1) was ultimately selected as the elution solvent. With this solvent, the elution efficiencies for aloe-emodin, rhein, emodin, chrysophanol, and physcion were 82.8%, 97.8%, 85.1%, 93.2%, and 65.3%, respectively.

#### 3.3.4. Adsorption Mechanism and Selectivity of ZIF-8 for Different Anthraquinones

The adsorption of anthraquinones onto ZIF-8 is governed by multiple synergistic interactions. First, the dominant driving force is π-π stacking between the aromatic rings of anthraquinones and the imidazole linkers of ZIF-8. Second, hydrogen bonding may form between the carbonyl (C=O) or hydroxyl (–OH) groups of anthraquinones and the nitrogen atoms in the ZIF-8 framework. Third, electrostatic interactions depending on solution pH and the pKa of anthraquinones also contribute to the adsorption. Finally, the microporous structure of ZIF-8 provides size-exclusion and confinement effects.

Notably, ZIF-8 exhibited different adsorption efficiencies toward individual anthraquinones, which can be explained by their structural differences. For example, aloe-emodin and rhein are likely to form intramolecular hydrogen bonds, resulting in reduced adsorption efficiency. In contrast, emodin and physcion exhibit lower elution efficiency. This can be attributed to the fact that the number and position of hydroxyl groups influence their hydrogen-bonding capacity, and more hydroxyl groups generally result in stronger adsorption. Additionally, the carboxyl group of rhein significantly increases its acidity, which weakens its electrostatic interaction with ZIF-8 and consequently reduces its adsorption performance. These results indicate that the adsorption is not only a physical process but is also governed by specific molecular recognition between ZIF-8 and each anthraquinone. Nevertheless, we acknowledge that the absence of adsorption kinetics and isotherm data in the present study constitutes a limitation, as such data would provide quantitative evidence for the adsorption rates and capacities. Systematic kinetic and isotherm investigations will be pursued in our future work to further validate the proposed adsorption mechanism and to better understand the molecular recognition process.

#### 3.3.5. Comparison with Previously Reported MOF-Based Extraction Systems

To further demonstrate the advantages of the proposed ZIF-8-based extraction method, we compared its performance with that of other MOF-based extraction systems reported in the literature for anthraquinones, structurally related aromatic compounds, or steroidal saponins. The comparison focused on four key parameters: amount of adsorbent, adsorption efficiency or recovery, extraction time, and limit of detection (LOD), as summarized in Table 4.

As shown in Table 4, our ZIF-8-based method achieved an adsorption efficiency of 80.2–100% for the five anthraquinones, which is comparable to previously reported values. More notably, the extraction time required for our method was only 10 min, which is significantly shorter than that reported for many other MOF-based systems. Furthermore, the LOD of our method is lower than most literature-reported values, indicating excellent sensitivity. Collectively, these comparisons clearly demonstrate that the proposed ZIF-8-based extraction method offers advantages in terms of time efficiency and detection sensitivity while maintaining high adsorption capacity.

### 3.4. Methodological Considerations

#### 3.4.1. Examination of Linear Relationship

To prepare the mixed standard solution, 1.80 mL of each standard stock solution was transferred into a 10 mL volumetric flask. The mixture was then diluted to the mark with methanol and thoroughly mixed. Subsequently, this mixed standard solution was diluted stepwise by factors of 2, 4, 8, 16, and 32, yielding six mixed standard solutions at different concentration levels. Each solution was injected under the chromatographic conditions, and each concentration level was measured three times in parallel. A standard curve was constructed using the average peak area from the three replicate measurements as the y axis and the reference substance concentration (x, µg/mL) as the x axis. The resulting linear regression equations, linear ranges, and correlation coefficients are summarized in Table 5.

#### 3.4.2. Limit of Detection and Limit of Quantification

The mixed reference solution that had been diluted 32-fold was further diluted in a gradient manner. The resulting solutions were then analyzed following the chromatographic conditions. The concentration giving a signal-to-noise ratio of 3:1 was taken as the limit of detection (LOD), and the concentration giving a signal to noise ratio of 10:1 was taken as the limit of quantification (LOQ). Table 5 presents the detailed results.

#### 3.4.3. Precision Test

The mixed standard solution diluted 4-fold was injected six times consecutively under the specified chromatographic conditions. The relative standard deviations (RSD) of the peak areas for aloe-emodin, rhein, emodin, chrysophanol, and physcion were calculated. The results showed RSD values of 0.3%, 0.3%, 0.4%, 0.3%, and 0.6%, respectively, indicating that the instrument exhibited good precision.

#### 3.4.4. Repeatability Test

Six samples from the same batch were processed using the sample preparation method described above. After processing, 20 mg of metal-organic frameworks (MOFs) were added to each sample for adsorption to obtain the test solutions. The solutions were then analyzed under the specified chromatographic conditions. The peak areas of aloe-emodin, rhein, emodin, chrysophanol, and physcion were recorded, and the adsorption efficiency of each component was calculated. The results showed that the RSD values of the adsorption efficiencies for all components were less than 3.0%, indicating that this method exhibits good repeatability.

#### 3.4.5. Stability Test

The test solution of Cassiae Semen that had been subjected to adsorption and elution with ZIF-8 NPs was analyzed at 0, 2, 4, 8, 12, and 24 h under the specified chromatographic conditions. The stability was assessed by examining changes in peak areas. The results showed that the RSD values of the peak areas for the five anthraquinones in Cassiae Semen were all below 4.0%, indicating that the test solution maintained good stability throughout the observed time period.

#### 3.4.6. Application to Real Samples

The dried Cassiae Semen sample was crushed and sieved through a 40 mesh screen. An accurately weighed portion of 0.1000 ± 0.0002 g of the powder was transferred into a stoppered Erlenmeyer flask. Then, 10 mL of 50% methanol solution was carefully added, and the total weight was recorded. The mixture was subjected to ultrasonic extraction at 50 °C for 20 min. After cooling, the lost weight was replenished with methanol, and the solution was thoroughly mixed. The resulting mixture was filtered through a 0.22 µm microporous membrane to obtain test solution a, which was subsequently analyzed under the described chromatographic conditions.

**Figure 10:**
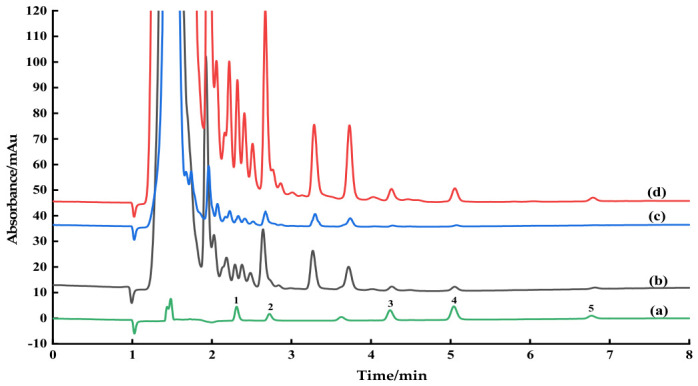
Chromatogram of Cassia semen extract: (a) Chromatogram of cassia semen extract; (b) Supernatant after MOFs adsorption of cassia semen extract; (c) Eluate after MOFs adsorption; (d) Standard mixture of five anthraquinones: 1. Aloe−emodin; 2. Rhein; 3. Emodin; 4. Chrysophanol; 5. Physcion.

A 0.0200 g portion of the as-prepared MOFs was accurately weighed and added into the previously described cassia semen extract. Then, the mixture was shaken for 10 min, followed by centrifugation at 9029× *g* for 10 min. The resulting supernatant was collected and filtered through a 0.22 µm microporous membrane to obtain test solution b. To the remaining precipitate, 1.00 mL of methanol/100 mM NaHCO_3_ (1:1, *v*/*v*) elution solvent was added. After sonication for 10 min, the mixture was centrifuged at 1000× *g* for 10 min, and the supernatant was retained as the eluted fraction, which was subsequently filtered through a 0.22 µm microporous membrane to yield test solution c. Both solutions were injected into the chromatograph under the aforementioned conditions for analysis, and the experimental outcomes are presented in Figure 10.

The experimental results demonstrated that ZIF-8, rapidly synthesized by the solvothermal method in this study, exhibited excellent selective adsorption properties toward all five target anthraquinone compounds. Except for aloe-emodin, which was significantly influenced by the matrix, the other four anthraquinones consistently showed strong selective adsorption behavior during the experimental process.

## 4. Conclusions

This study systematically optimized synthesis conditions including solvent, material ratio, reaction time, and reaction temperature, leading to the successful preparation of ZIF-8 NPs. The obtained material demonstrates excellent adsorption capabilities for five anthraquinones, namely aloe-emodin, rhein, emodin, chrysophanol, and physcion. The preparation procedure is both straightforward and efficient. Furthermore, by optimizing additional parameters such as adsorbent dosage, adsorption time, and the composition of the elution solvent, the material’s adsorption performance toward these target compounds was further enhanced. Under the optimized conditions, the adsorption rates ranged from 80.2% to 100%, while the elution efficiencies were between 65.3% and 97.8%. Considering the excellent performance of the UPLC method in terms of linear range, limits of detection (LODs), and precision, it can be concluded that the prepared ZIF-8 NPs are suitable for the selective extraction of these five anthraquinones from Cassia semen. Furthermore, this established adsorption and elution strategy based on ZIF-8 nanoparticles shows good application potential. It can be readily extended to the pretreatment and analysis of anthraquinones in other medicinal plants including rhubarb, fleece-flower root, aloe, and senna leaf, which greatly expands its application range.

The chemical composition of Cassiae Semen is complex, containing not only the five aforementioned common anthraquinones but also other bioactive components such as cassiaside, obtusin, aurantio-obtusin, cassic acid, and cassialactone. Therefore, in addition to optimizing the chromatographic conditions, this study also investigated several key parameters for sample pretreatment, including the type of extraction method, extraction solvent, temperature, and extraction time. By comparing the chromatographic peak areas of the target compounds obtained with different extraction solvents, a 50% methanol solution was ultimately selected as the optimal extraction solvent, and ultrasonic extraction was carried out at 50 °C for 20 min.

During the application of the as-prepared MOFs for the adsorption and separation of five anthraquinones from Cassia semen extract, it was observed that this material also effectively adsorbed two unknown peaks with retention times of 3.28 min and 3.73 min. These peaks are speculated to correspond to other anthraquinone active components present in Cassia semen, such as obtusifolin, obtusin, or chryso-obtusin. However, confirmation of their identities could not be achieved due to the lack of corresponding reference standards.

In addition, aloe-emodin was markedly influenced by the matrix, while physcion exhibited relatively low elution efficiency. Future research should focus on optimizing the chromatographic conditions including replacing the chromatographic column, adjusting the mobile phase composition, and modifying the gradient elution program to enhance separation efficiency and reduce matrix interference for aloe-emodin, as well as further improving the elution conditions to increase the elution recovery of physcion. To this end, we propose using a stronger alkaline reagent, such as sodium carbonate or sodium hydroxide, to improve the elution recovery of physcion. The rationale is that physcion, as an anthraquinone compound with a phenolic hydroxyl group, can be converted into its water-soluble salt form under strongly alkaline conditions, thereby significantly enhancing its desorption from the adsorbent and its solubility in the elution solvent. After elution, the pH can be carefully adjusted to weakly acidic conditions to recover physcion in its original form. In addition, the volume of elution solvent and the elution time can also be increased. These strategies are expected to substantially increase the elution efficiency of physcion, and will be systematically evaluated in our future work.

## Figures and Tables

**Table 1:** Levels and code of reaction variables used in Box–Behnken design.

Variable	Coded Levels		
	−1	0	1
Reaction temperature (A, °C)	60	120	180
Material ratio (molar ratio of 2-methylimidazole to zinc acetate, B)	3	5	7
Reaction time (C, h)	2	4	6

**Table 2:** Box–Behnken experimental design and the results for the adsorption efficiency of ZIF-8 toward five anthraquinones.

Run	Factors			Adsorption Efficiency (%)				
	A	B	C	Aloe-Emodin	Rhein	Emodin	Chrysophanol	Physcion
1	60	3	4	63.3	70.8	82.1	83.4	81.2
2	180	3	4	67.9	78.6	86.8	87.5	84.5
3	60	7	4	66.8	72.3	82.3	83.5	80.2
4	180	7	4	72.6	83.1	92.3	91.3	90.8
5	60	5	2	66.5	71.4	82.3	83.1	80.7
6	180	5	2	71.8	82.6	91.2	90.3	89.6
7	60	5	6	65.7	71.2	82.5	83.2	80.9
8	180	5	6	71.2	81.6	91.1	90.7	90.5
9	120	3	2	74.5	84.3	90.3	89.5	87.3
10	120	7	2	75.1	84.8	92.1	92.7	91.5
11	120	3	6	72.8	82.6	90.4	90.1	88.7
12	120	7	6	76.2	86.3	92.5	92.8	91.1
13	120	5	4	80.2	93.8	100	100	100
14	120	5	4	80.6	92.5	98.9	98.2	97.8
15	120	5	4	81.5	94.9	99.6	98.7	98.5
16	120	5	4	79.8	93.2	99.3	99.6	99.1
17	120	5	4	78.7	94.6	98.6	99.3	99.7

**Table 3:** ANOVA of response surface model and predicted results for response of five anthraquinones.

Source	Aloe-Emodin		Rhein		Emodin		Chrysophanol		Physcion	
	*F*-Value	*p*-Value	*F*-Value	*p*-Value	*F*-Value	*p*-Value	*F*-Value	*p*-Value	*F*-Value	*p*-Value
Model	62.73	<0.0001	161.40	<0.0001	196.81	<0.0001	129.30	<0.0001	101.66	<0.0001
A	59.98	0.0001	254.55	<0.0001	344.56	<0.0001	175.04	<0.0001	151.94	<0.0001
B	19.86	0.0029	16.39	0.0049	30.63	0.0009	23.76	0.0018	20.50	0.0027
C	0.5338	0.4887	0.3087	0.5958	0.1196	0.7396	0.3562	0.5694	0.6383	0.4506
AB	0.3843	0.5549	2.84	0.1361	18.67	0.0035	6.77	0.0353	15.43	0.0057
AC	0.0107	0.9206	0.2016	0.6670	0.0598	0.8138	0.0445	0.8389	0.1418	0.7176
BC	2.09	0.1913	3.23	0.1155	0.0598	0.8138	0.1237	0.7354	0.9379	0.3651
A^2^	378.80	<0.0001	855.77	<0.0001	902.18	<0.0001	615.23	<0.0001	443.24	<0.0001
B^2^	49.84	0.0002	127.39	<0.0001	219.43	<0.0001	143.00	<0.0001	137.46	<0.0001
C^2^	21.36	0.0024	102.72	<0.0001	139.29	<0.0001	116.71	<0.0001	80.36	<0.0001
Lack of Fit	0.7228	0.5888	0.5658	0.6661	1.53	0.3375	0.4950	0.9649	0.9525	1.20
R^2^	0.9878		0.9952		0.9961		0.9940		0.9924	
R^2^ _Adj_	0.9720		0.9890		0.9910		0.9863		0.9826	
R^2^ _Pred_	0.9187		0.9719		0.9635		0.9544		0.9363	
Adeq Precision	22.0733		33.8830		37.3206		30.0082		26.9832	

**Table 4:** Comparison of the proposed ZIF-8-based SPE-UPLC method with previously reported MOF-based extraction systems.

Method	MOF Material	Target Analytes	Adsorbent Amount (mg)	Adsorption Efficiency/Recovery (%)	Extraction Time (min)	LOD (ng/mL)	Reference
SPE-UV	Cu(II)-MOF	Quercetin(QT)	5	98.8	1440	–	[18]
SPE-HPLC	ZIF-8	3,4-dihydroxy-8,9-methylenedioxypterocarpan	1	62.1–75.4	90	–	[19]
MSPD-UPLC/MS	MOF-808	ginsenosides Rg2, Rg1, Rb1, Re, and Rd	20	87.0–103.7	60	87–114	[20]
MSPE-HPLC	MIL-101(Fe)	Aloe-emodin, emodin, and physcion	20	92.3–116.1	20	1.7–3.4	[23]
SPE-UPLC	ZIF-8	Aloe-emodin, rhein, emodin, chrysophanol, and physcion	20	80.2–100	10	30–63	This work

**Table 5:** Standard curves, linear ranges, detection limits, and quantification limits for 5 types of anthraquinones.

Component Name	Regression Equation	Linear Range (µg/mL)	Correlation Coefficient (r)	Detection Limit (µg/mL)	Quantification Limit (µg/mL)
Aloe-emodin	Y = 0.1465X − 0.0013	0.650–20.808	1.0000	0.033	0.098
Rhein	Y = 0.1185X − 0.0134	0.534–17.388	0.9997	0.053	0.160
Emodin	Y = 0.1571X + 0.0279	0.658–21.060	1.0000	0.033	0.099
Chrysophanol	Y = 0.2151X + 0.0526	0.604–19.332	1.0000	0.030	0.091
physcion	Y = 0.0458X + 0.0070	0.507–16.236	0.9997	0.063	0.144

## Data Availability

The original contributions presented in this study are included in the article. Further inquiries can be directed to the corresponding authors.

## Abbreviations

The following abbreviations are used in this manuscript:

RSM	Response Surface Methodology
BBD	Box–Behnken design
MOFs	Metal Organic Frameworks
ZIFs	Zeolitic Imidazolate Frameworks
NPs	Nanoparticles
SEM	Scanning Electron Microscope
TEM	Transmission Electron Microscope
UPLC	Ultra Performance Liquid Chromatography
RSDs	Relative Standard Deviations
LODs	Limits of Detection
